# Revisiting Japan's early COVID‐19 countermeasures

**DOI:** 10.1002/hsr2.1468

**Published:** 2023-07-28

**Authors:** Yudai Kaneda, Yuka Higuchi, Kenzo Takahashi

**Affiliations:** ^1^ School of Medicine Hokkaido University Hokkaido Japan; ^2^ Teine Keijinkai Hospital Hokkaido Japan; ^3^ Teikyo University Graduate School of Public Health Tokyo Japan

Three years have elapsed since the onset of the pandemic, and in Western countries and scientific journals, a retrospective evaluation and reflection on the measures taken against the Coronavirus Disease 2019 (COVID‐19) are being conducted scientifically. In the early stages of the emergence of COVID‐19, there was limited information available and no vaccine yet, which posed challenges for governments in devising effective strategies, implementing policies, and demonstrating leadership. Indeed, a report submitted to the World Health Assembly in 2021 highlighted the inadequacy of risk communication, ineffective planning, and delays in response in addressing COVID‐19.^1^ In contrast, the attitude of the Japanese government and its officially appointed experts, as represented by their reports in scientific journals, can so far be described as questionable.

Specifically, in a study in which some of the authors were members of the Japanese government's Subcommittee on Novel Coronavirus Disease Control, Imamura et al. have reported that prioritizing surveillance and intervention in nightlife settings was effective in preventing the spread of COVID‐19, particularly in the early stages of the outbreak.^2^ This conclusion was based on the surveillance of infection rates in Tokyo from January 23 to December 5, 2020, when vaccinations had not yet been administered.^2^ However, during this investigation period, PCR testing was limited to those with severe symptoms, inevitably overlooking asymptomatic individuals or those with mild symptoms. Consequently, relying on the reported data from Japan during this period may result in an underestimation of the number of infections. Furthermore, the study posits a hypothesis that super spreading events (SSE), events characterized by numerous secondary infections initiated by super‐spreaders, individuals who are more likely to transmit to others than the otherwise standard infected person, spreading infections much more than usual, contribute to the expansion of infections, which is based on a modeling study by Endo et al.^3^ Nevertheless, this paper concludes without accurately examining the impact of SSE on the occurrence, and to the best of our knowledge, there is no evidence to support the claim that SSE caused the spread of infections. Taking these points into consideration, it is possible that the authors' data sampling methods were biased toward affirming the policies of the time. Therefore, caution is necessary when interpreting this study's conclusion that prioritizing monitoring and intervention in nightlife environments was warranted.

A similar study has also been reported in the past. An officially appointed expert in Japan noted that Japan had minimized the damage compared to the G7 countries by promptly applying the knowledge of aerosol transmission of COVID‐19 to countermeasures and providing information more effectively than Western countries, and as of May 2022, the infection was almost under perfect control.^4^ Nonetheless, a study assessing excess mortality between January 2020 and December 2021 found that Japan had the highest excess mortality rate among OECD nations, with an estimated 111,000 deaths—a figure that is six times greater than the officially confirmed COVID‐19 death count of 18,400.^5^ This calls into question the scientific validity of the assessment mentioned above. In this background, we surmise that there was a failure by the government, as they implemented cluster control measures mainly at public health centers to track the route of infection, especially among persons in closely contacted with infected persons, from the mistaken viewpoint that COVID‐19 differs from influenza viruses in that about 80% of infected persons would not transmit the virus to others, regardless of severe or mild symptoms, and that the spread of infection occurs mainly through the formation of clusters. Moreover, the fact of airborne transmission of COVID‐19 was also never considered in policymaking until more than a year after it was reported in scientific journals.^6^ These factors may have led to the tragic excess mortality.

In conclusion, considering the situations described above, politicians and the experts consulted have yet to assess Japan's approach to COVID‐19 properly. The oversight of aerosol transmission was a misstep shared by public health organizations, including the WHO, which indicates that it may have been an inescapable issue in the realm of international collaboration.^6^ However, unless there is a demonstrated willingness to reflect on and learn from the shortcomings of early COVID‐19 responses in the context of international cooperation, there is a risk of further problems and potentially leading to a prolonged public health crisis, as highlighted by the potential threat of new infectious diseases such as avian influenza and expected other novel infectious diseases. The Japanese government and experts must reevaluate their past COVID‐19 policies, considering the lessons learned from domestic and international experiences and emphasizing scientific credibility.

## AUTHOR CONTRIBUTIONS

**Yudai Kaneda**: Conceptualization; data curation; writing—original draft. **Yuka Higuchi**: Conceptualization; data curation; writing—original draft. **Kenzo Takahashi**: Writing—review and editing.

## CONFLICT OF INTEREST STATEMENT

The authors declare no conflict of interest.

## TRANSPARENCY STATEMENT

The lead author (Yudai Kaneda) affirms that this manuscript is an honest, accurate, and transparent account of the study being reported; that no important aspects of the study have been omitted; and that any discrepancies from the study as planned (and, if relevant, registered) have been explained.

## Data Availability

Data available on request from the authors.
